# Values in Romantic Relationships

**DOI:** 10.1177/01461672231156975

**Published:** 2023-03-21

**Authors:** Reine C. van der Wal, Lukas F. Litzellachner, Johan C. Karremans, Nadia Buiter, Jamie Breukel, Gregory R. Maio

**Affiliations:** 1 Utrecht University, The Netherlands; 2 University of Bath, UK; 3 Radboud University, Nijmegen, The Netherlands

**Keywords:** values, romantic relationship functioning, relationship quality, attitudes

## Abstract

There are substantive theoretical questions about whether personal *values* affect romantic relationship functioning. The current research tested the association between personal values and romantic relationship quality while considering potential mediating mechanisms related to pro-relational attitudes, communal strength, intrinsic relationship motivation, and entitlement. Across five studies using different measures of value priorities, we found that the endorsement of self-transcendence values (i.e., benevolence, universalism) was related to higher romantic relationship quality. The findings provided support for the mediating roles of pro-relational attitudes, communal strength, and intrinsic relationship motivation. Finally, a dyadic analysis in our fifth study showed that self-transcendence values mostly influence a person’s own relationship quality but not that of their partner. These findings provide the first evidence that personal values are important variables in romantic relationship functioning while helping to map the mechanisms through which this role occurs.

When people struggle with their romantic relationships, it is easy to find fault with their partner. In our humbler moments, we might also recognize contributions from our own traits and habits. But what about the potential impact of our own cherished personal values? Dating websites frequently mention the importance of values in finding the right match, and recent research has reported that overt value disagreements are significant challenges in partners who take opposed stances on contentious political issues (Afifi et al., 2020). Yet, the role of personal values per se in general relationship functioning has escaped attention. Could it be the case that some types of values imperil our chances of success at happiness in our romantic relationships?

In recent decades, personal values have received a great deal of attention in the context of other scientific problems. Often defined as cognitive representations of abstract goals that serve as *guiding principles in life* (Rokeach, 1973; Schwartz, 1992), personal values predict attitudes, opinions, decisions, and behavior in various life domains (for an overview, see Maio, 2017; Roccas & Sagiv, 2010). However, relationship scientists have devoted little attention to the potential role of personal values in romantic relationships. There is research showing that romantic relationship partners share similar values (Watson et al., 2004) and that the extent to which they share the same values promotes interpersonal attraction (Lee et al., 2009; Sprecher & Regan, 2002), perhaps with implications for relationship satisfaction (Leikas et al., 2018). Nevertheless, it remains unknown whether and how the *content* of people’s values affects their romantic relationships. Does it really matter whether I value creativity or family security? Does it make a difference whether I prioritize helping others or wealth? In the current research, we examine the role of personal values in romantic relationship quality and the mechanisms that underpin these associations.

The most widely used model of human values is Schwartz’s (1992) Value Theory, which predicts a structure of values that has been found in more than 75 countries worldwide (Schwartz, 2011). The model was revised in 2012 to include 19 motivationally distinct types of values and specifies the dynamic relations among them (Figure 1; Schwartz et al., 2012). On a higher level, the 19 values fall into two dimensions, where each oppositional pole reflects opposing motivations: self-enhancement (i.e., dominance over others and pursuit of personal success) versus self-transcendence (i.e., acceptance of others and concern for their welfare), and openness to change (i.e., readiness for change and independent thought and action) versus conservation (i.e., preserving tradition and protecting stability).

**Figure 1.**
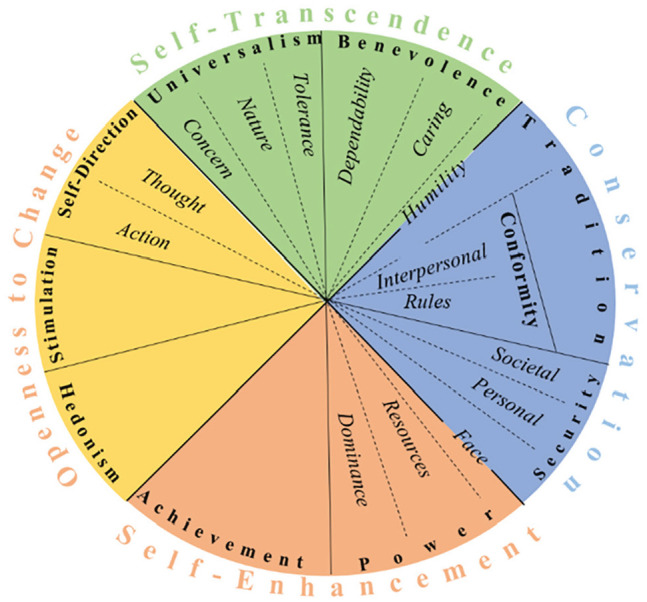
Theoretical Model of the Structure of Relations Among 19 Values (Schwartz et al., 2012).

These value dimensions are relevant to romantic relationships because relationship well-being and functioning are determined by interpersonal interactions within a relationship. In general, romantic relationships are characterized by more stability and higher quality when partners exhibit a strong commitment to each other (Rusbult et al., 1998) and show behavior that promotes mutual trust (Wieselquist et al., 1999). Such behaviors might include accommodation (Rusbult et al., 1991), sacrifice (Van Lange et al., 1997), capitalization (Reis et al., 2010), and having a communal mindset, in which partners focus on the needs of the other and in which direct reciprocity is not the norm (Mills et al., 2004). We propose that these diverse relationship processes and behaviors partly originate from a broader set of values that people have. In that sense, values may be a relatively distal factor in contributing to relationship functioning. On the contrary, the processes and behaviors that occur in a relationship may for an important part be driven by values, and as such, values may actually play a crucial role in shaping relationship well-being. This is an empirical question, which has received very little attention.

Specifically, we expect that the endorsement of self-transcendence values, which motivate acting for the benefit of others, helps to focus on the partner and on the relationship, resulting in better relationships. On the contrary, we expect that the endorsement of self-enhancement values, which motivate acting for the benefit of oneself, undermines this focus on the partner and on the relationship, resulting in worse relationship outcomes. This reasoning is supported by previous findings. Self-transcendence values have been portrayed as “guiding principles of interpersonal behavior” (Mikulincer et al., 2010), and satisfaction with social life is often characterized by the endorsement of self-transcendence values (Schwartz & Sagiv, 1995). Self-transcendence values also positively relate to forgiveness (Strelan et al., 2011). Moreover, relationship partners who highly endorse power (i.e., self-enhancement) values tend to be less motivated to sacrifice for and give support to their partner (Righetti et al., 2015; Van Willigen & Drentea, 2001). Such partners also place less value on the support they receive from their partner (Inesi et al., 2012). Following this line of thought, we expected that self-transcendence values would be positively related to romantic relationship quality, and self-enhancement values would be negatively related to romantic relationship quality.^1^

It is also theoretically interesting whether the other dimension—openness to change values versus conservation values—is associated with romantic relationships. On one hand, it could be that openness to change values are associated with processes that prevent relationship boredom, which seems to be crucial in romantic relationships (Tsapelas et al., 2009). On the other hand, it could be that the endorsement of conservation values corresponds with the motivation to maintain the relationship because they pay less attention to attractive alternatives (Simpson & Gangestad, 1990). Given these possibilities, it is unclear whether and how this value dimension is associated with relationship quality. We explore this association in the current research.

## The Present Research

In all our studies, we take a broad view of relationship quality, construing it as an intraindividual relationship perception, consisting of multiple components (i.e., relationship satisfaction, commitment, intimacy, trust, passion, and love; Fletcher et al., 2000b). Conceptualized as a multifaceted construct, relationship quality has been shown to link to many important relationship outcomes such as relationship stability and lower chances of relationship dissolution (Fletcher et al., 2000a). This conceptualization makes our results also more comparable with other research, as the most prominent measure of multifaceted relationship quality, the Perceived Relationship Quality Component (PRQC; Fletcher et al., 2000a) has been cited more than 1,000 times. Accordingly, utilizing measurements associated with this definition allows us to gain insight into the effects of broad motivational value dispositions on relationship functioning.

To provide a robust starting point for our research, we started with an online study (Study 1) examining associations between value priorities and romantic relationship quality. In Study 1, we measured people’s value priorities using the 57-item Portrait Values Questionnaire (PVQ; Schwartz et al., 2012). Because we did not want our findings to rely on one specific values measure only, we tested the same associations in Study 2 but using the Short Schwartz Value Survey (SSVS; Lindeman & Verkasalo, 2005). In Study 3, we examined whether pro-relationship attitudes (i.e., attitudes toward forgiveness, (in)fidelity, and sacrifice) mediate associations between values and relationship quality. In Study 4, we pre-registered and replicated Studies 1 to 3 and additionally explored mediators reflecting behavior in romantic relationships (i.e., communal strength, intrinsic relationship motivation, and entitlement). In Study 5, we adopted a dyadic approach and examined actor and partner effects of values on relationship quality in heterosexual couples. In all analyses, we took into account relationship duration to control for the honeymoon effect (Lorber et al., 2015), which is the tendency for individuals who have recently coupled to experience greater relationship satisfaction than individuals who have been together longer. Moreover, as age is associated with value priorities (Robinson, 2013), we controlled for age in our analyses. All data and analysis scripts can be viewed at the Open Science Framework (https://rb.gy/yonx41).

## Study 1

Study 1 was designed to provide initial evidence for our hypothesis that value priorities are associated with romantic relationship quality.

### Method

#### Participants and Design

We recruited 248 participants through Amazon’s Mechanical Turk (MTurk) in exchange for $1.00. Eleven participants were excluded from the analyses because they failed an instructed-response attention check (Kung et al., 2018). The final sample consisted of 237 participants (126 women) aged 20 to 74 (*M* = 36.75, *SD* = 11.75). Most of the participants were Caucasian (85%), the rest were Hispanic (4%), Black or African American (5%), Asian or Pacific Islander (4%), or Other (1%). Most participants had a college or university degree (57%) or some college education (28%). All participants were currently involved in a romantic relationship, mostly heterosexual (*N* = 218), with a duration of 0 to 52 years (*M* = 9.95, *SD* = 10.40). Most participants (*N* = 106) had at least one child with their current partner. A post hoc sensitivity analysis (α = .05, 80% power) showed that our sample was large enough to detect small to medium-sized correlations (*r* = .18; G*Power, Version 3.1.9.2, Faul et al., 2009).

#### Procedure

The data were collected using Qualtrics software. After giving informed consent, participants were instructed that they would receive several questionnaires tapping into value priorities and relationship quality. Participants were allowed to quit the survey at any point. The software allowed any question to be left blank, but it did not allow participants to return to previous screens to change responses. After completing the survey, participants were thoroughly debriefed and thanked.

#### Materials

##### Values

We used separate gender-matched versions of the PVQ value survey, consisting of 57 value items presented in randomized order (Schwartz et al., 2012). Participants were asked to rate fictitious people in terms of similarity to themselves. Example items were “Caring for the well-being of people he or she is close to is important to him or her” (self-transcendence), “he or she thinks it is important to be ambitious” (self-enhancement), “he or she is always looking for different kinds of things to do” (openness to change), and “he or she strongly values the traditional practices of his or her culture” (conservation). Answers were given on a 6-point scale from 1 (*not like me at all*) to 6 (*very much like me*). Each higher order value domain (self-transcendence, self-enhancement, openness to change, and conservation) showed acceptable reliability consistent with prior evidence for the PVQ (.77 < Cronbach’s α < .90). To keep each domain as clearly defined as possible, items that can overlap with more than one domain were excluded from the analysis (i.e., Humility, Face, and Hedonism; Schwartz et al., 2012; but see Table S2 in the supplemental materials for the results including these values). As recommended by Schwartz (2016), we used uncentered mean-level responses, as our regression analysis entered all value types simultaneously (see also Borg & Bardi, 2016; see Table S4 in the supplemental materials for correlation results using centered scores).

##### Relationship Quality

We measured participants’ relationship quality with the PRQC (Fletcher et al., 2000). Responses were given on a 7-point Likert-type scale, ranging from 1 (*do not agree at all*) to 7 (*agree completely*); *M* = 5.80, *SD* = 1.12, Cronbach’s α = .95.

### Results

To examine the unique associations between value priorities and relationship quality, we conducted linear regression analyses in which we regressed relationship quality on the four higher order dimensions of values (i.e., self-transcendence, self-enhancement, openness to change, and conservation), controlling for relationship duration and age. The results showed that individuals who highly valued self-transcendence exhibited higher relationship quality (Table 1). Contrary to our expectations, valuing self-enhancement was not related to lower relationship quality. Neither openness to change nor conservation values significantly predicted relationship quality. Results remained essentially the same when not controlling for relationship duration and age (Table S1 in the supplemental materials).

**Table 1.** Regression of Relationship Quality With Uncentered Value Priorities Including Relationship Duration and Age (Studies 1–5).

	Study 1: Relationship quality by PVQ (*N* = 237)					Study 2: Relationship quality by SSVS (*N* = 222)					Study 3: Relationship quality by PVQ (*N* = 233)				
Variables	β	CI_low_	CI_high_	*t*	*p*	β	CI_low_	CI_high_	*t*	*p*	β	CI_low_	CI_high_	*t*	*p*
Self-transcendence	0.20	0.05	0.39	2.55	.012	0.25	0.08	0.42	2.94	.004	0.18	0.01	0.33	2.13	.034
Self-enhancement	−0.11	−0.29	0.04	−1.49	.138	−0.01	−0.13	0.15	0.23	.827	−0.11	−0.25	0.03	−1.61	.110
Openness to change	−0.03	−0.21	0.14	−0.43	.669	−0.12	−0.27	0.04	−1.43	.154	0.28	0.13	0.43	3.64	<.001
Conservation	0.15	0.02	0.33	2.16	.031	0.08	−0.08	0.24	0.95	.341	0.08	−0.06	0.23	1.14	.254
Relationship duration	0.18	0.00	0.41	1.97	.050	0.23	0.03	0.43	−2.22	.027	0.02	−0.12	0.16	0.30	.764
Age	−0.31	−0.56	−0.14	−3.24	.001	−0.38	−0.59	−0.17	3.59	<.001	0.03	−0.11	0.17	0.40	.690
	*F*(6, 228) = 3.83, *p* = .001, *R*^2^ = .09					*F*(6, 216) = 2.58, *p* = .029, *R*^2^ = .03					*F*(6, 225) = 7.89, *p* < .001, *R*^2^ = .17				
	Study 4: Relationship quality by PVQ (*N* = 310)					Study 4: Relationship quality by SSVS (*N* = 288)					Study 5: Relationship quality by SSVS (*N* = 562 couples)				
	β	CI_low_	CI_high_	*t*	*p*	β	CI_low_	CI_high_	*t*	*p*	β	CI_low_	CI_high_	*t*	*p*
Self-transcendence	0.22	0.08	0.35	3.05	.003	0.24	0.10	0.34	3.58	<.001	0.13	0.06	0.19	3.64	<.001
Self-enhancement	−0.12	−0.24	0.01	−1.84	.066	0.02	−0.09	0.13	0.36	.719	0.02	−0.04	0.08	0.66	.510
Openness to change	0.08	−0.06	0.22	1.07	.285	0.004	−0.11	0.12	0.06	.951	−0.03	−0.10	0.03	−1.05	.296
Conservation	0.10	−0.03	0.22	1.49	.136	0.10	−0.02	0.22	1.64	.103	0.02	−0.04	0.08	0.59	.557
Relationship duration	0.05	−0.11	0.20	0.61	.542	0.025	−0.13	0.18	0.29	.771	−0.05	−0.15	0.05	−1.05	.328
Age	−0.18	−0.32	−0.02	−2.19	.029	−0.17	−0.32	−0.003	−2.00	.046	−0.07	−0.16	0.02	−1.54	.124
	*F*(6, 303) = 5.87, *p* < .001, *R*^2^ = .10					*F*(6, 281) = 4.47, *p* < .001, *R*^2^ = .09					*F*(6, 1117) = 5.00, *p* <.001, *R*^2^ = .02				

*Note.* PVQ = Portrait Values Questionnaire; SSVS = Short Schwartz Value Survey.

### Discussion

The findings of Study 1 provide initial evidence for our reasoning that people’s value priorities are associated with their romantic relationship quality. As expected, prioritizing self-transcendence values was significantly associated with higher relationship quality. Interestingly, prioritizing self-enhancement values was not associated with lower relationship quality. Potentially, this pattern of results implies that the presence of prosocial motives is more beneficial for relationship quality than the presence or absence of self-focused motives (cf. Visserman et al., 2018). The extent to which people endorse openness to change values or conservation values was unrelated to relationship quality.

## Study 2

To check the robustness of our findings, we aimed to replicate them using a different measure of personal values. Roccas et al. (2017) categorize measures of personal values as either concrete or abstract. In a concrete measure, participants do not rate the importance of abstract values, but their similarity to a fictitious individual who expresses a pattern of thought or behavior, related to a value. Roccas et al. describe the PVQ (which we used in Study 1) as an example of a concrete measure. In an abstract measure, participants directly rate the importance of the abstract principles in their lives (e.g., “How important is *equality* as a guiding principle in your life”). Prime examples of abstract instruments are the Schwartz’s Value Survey (Schwartz, 1992) and its short form, the Short Schwartz’s Value Survey (SSVS) (Lindeman & Verkasalo, 2005). We sought to replicate our findings from the PVQ used in Study 1 with a more abstract instrument in Study 2.

### Method

#### Participants and Design

As part of a larger study (Litzellachner et al., 2023), we recruited 260 individuals in romantic relationships via Prolific Academic in exchange for £1.50. We excluded 29 participants for failing an instructed-response attention check (Kung et al., 2018). The final sample comprised 231 individuals (105 men, 121 women, 5 NAs, *M*_age_ = 32.52, *SD*_age_ = 10.39). The average relationship duration was 8.37 years (*SD* = 8.33) ranging from 1 month to 51 years (nine individuals did not submit their relationship duration). Almost half of all relationships were dating relationships (56%), the other half being marriages (44%). Most relationships were heterosexual (96%). Only a minority of participants (34%) indicated having a child with their partner. The majority of our responses were from European countries (87%), and most of the participants were currently in employment (54%), self-employment (12%), or students (19%). According to a post hoc sensitivity analysis (α = .05, 80% power), our sample size was sufficient to detect small- to medium-sized correlations (*r* > .18; G*Power, Version 3.1.9.2, Faul et al., 2009).

#### Procedure

All questionnaires were created using Qualtrics. Participants first received either a block of questionnaires with relationship instruments or a block of questionnaires with the predictor instruments. The relationship block contained two measures of relationship quality (the PRQC and a single-item measure of relationship happiness), a single-item measure of commitment, and a measure of cognitive interdependence. The predictor block consisted of the SSVS, a measure of perceived value similarity with their partner, and ideographic questions about personal goals and partner supportiveness. While the order of the blocks was randomized, the order of the questionnaires within the blocks was fixed. Below, we describe in detail only the measures relevant to this article, the SSVS and the measure of relationship quality, and not those that were part of the aforementioned larger study (Litzellachner et al., 2023).

#### Materials

##### Values

Participants rated the importance of the 19 value types from Schwartz’s refined theory of basic values (Schwartz et al., 2012). Participants received the name of the value type (e.g., “Hedonism,” “Self-Direction Action”) accompanied by a short description (e.g., “Pleasure and sensuous gratification,” “Freedom to determine one’s action”). These descriptions were slightly shortened versions of the value-type descriptions given by Schwartz et al. (2012). Participants rated the importance of the values as guiding principles in their lives on a scale from −1 (*opposed to my values*) to 7 (*of supreme importance*). The internal consistencies for self-enhancement (Cronbach’s α = .79) and self-transcendence (Cronbach’s α = .76) were good, but weaker for openness and conservation (both Cronbach’s α = .67), as seen in other work (e.g., Schwartz et al., 2014). As in Study 1, we excluded values that overlapped multiple higher-order value dimensions (but see Table S2).

##### Relationship Quality

The PRQC (Fletcher et al., 2000b) used in this study was identical to the one used in Study 1 and had high internal consistency (Cronbach’s α = .97, *M* = 5.69, *SD* = 1.06).

### Results

All uncentered value scores were simultaneously entered into a linear regression model predicting relationship quality. The results show that individuals who highly valued self-transcendence exhibited higher relationship quality, replicating previous findings (Table 1). Against our expectations, valuing self-enhancement was not related to lower relationship quality. Neither openness nor conservation values showed significant relations with relationship quality.

### Discussion

We replicated our previous findings using a different value measure, showing that higher self-transcendence relates to higher relationship quality. The finding was obtained using the SSVS, which is much shorter and more abstract than the PVQ (Roccas et al., 2017). Thus, the prediction of relationship quality from self-transcendence values is robust across the distinct measures of values used in Study 1 and Study 2.

## Study 3

In Study 3, we sought to replicate and extend the findings by examining the associations between value priorities, relationship quality, and attitudes toward several pro-relationship behaviors. By examining these associations, we were able to examine a mechanism implied by past research on values, attitudes, and behavior. Specifically, it has been proposed that attitudes arise out of basic personal values and are expressed through what people say and do (Boer & Fischer, 2013; Maio & Olson, 1995), reflecting a long line of research on the psychological functions of attitudes (Maio & Olson, 2000). These attitudes, in turn, are proximal predictors of decisions and behaviors (e.g., Homer & Kahle, 1988; see Maio, 2017). Consequently, we expected that the values people endorse may be connected to the quality of romantic relationships through the way in which people evaluate behavior in the context of their romantic relationships (i.e., attitudes toward pro-relationship behavior). We therefore expected that people who endorse more self-transcendence values have higher quality romantic relationships because they hold more positive attitudes toward different types of pro-relationship behaviors, such as forgiveness, (in)fidelity, and sacrifice.

### Method

#### Participants and Design

We recruited 243 American adults currently involved in romantic relationships through Amazon’s MTurk in exchange for $1.50. Ten participants were excluded from the analyses because they failed an instructed-response attention check (Kung et al., 2018). The final sample consisted of 233 participants (104 men; 129 women) between the ages of 19 and 73 (*M* = 37.15, *SD* = 11.51). Most of the participants were Caucasian (86%), the rest were Hispanic (3%), Black or African American (5%), Asian or Pacific Islander (4%), or Other (1%). Most participants had either a college or university degree (44%), or some college education (40%). Most of the participants’ relationships were heterosexual (88%), with a duration of 0 to 53 years (*M* = 8.54, *SD* = 9.34). Most commonly, participants had at least one child with their current partner (47%). A post hoc sensitivity analysis (α = .05, 80% power) showed that our sample was large enough to detect small to medium correlations (*r* = .18; G*Power, Version 3.1.9.2, Faul et al., 2009).

#### Procedure

The procedure was similar to Study 1, with data collected using Qualtrics software. After giving informed consent, participants were instructed that they would receive several questionnaires tapping into value priorities, relationship quality, and relationship attitudes. After completing the survey, participants were thoroughly debriefed and thanked.

#### Materials

##### Values

As in Study 1, we used separate gender-matched versions of the 57-item PVQ value survey (Schwartz et al., 2012) and excluded domain-overlapping items. Each higher order value domain (self-transcendence, self-enhancement, openness to change, and conservation) showed good reliability (.83 < Cronbach’s α < .89).

##### Relationship Quality

The relationship quality scale was identical to the one used in Study 1. It showed excellent reliability (*M* = 5.94, *SD* = 0.99, Cronbach’s α = .95).

##### Pro-Relationship Attitudes

The participants’ attitudes toward forgiveness were measured using the six-item scale developed by Brown (2003; e.g., “It is admirable to be a forgiving person”). Responses were given on a 7-point Likert-type scale, ranging from 1 (*strongly disagree*) to 7 (*strongly agree*); *M* = 4.94, *SD* = 1.01, Cronbach’s α = .75.

The participants’ attitudes toward infidelity were measured using the 12-item scale developed by Whatley (2008; e.g., “It would be acceptable for me to be unfaithful to my partner”). Responses were given on a 7-point Likert-type scale, ranging from 1 (*strongly disagree*) to 7 (*strongly agree*); *M* = 1.95, *SD* = 0.83, Cronbach’s α = .86.

The participants’ attitudes toward sacrifice were measured using the six-item Satisfaction with Sacrifice Scale (Stanley & Markman, 1992; e.g., “It makes me feel good to sacrifice for my partner”). Responses were given on a 7-point Likert-type scale, ranging from 1 (*strongly disagree*) to 7 (*strongly agree*); *M* = 5.69, *SD* = 1.12, Cronbach’s α = .92.

### Results

The four uncentered value scores were simultaneously entered into a linear regression model predicting relationship quality, thereby controlling for relationship duration. In line with the findings from Studies 1 and 2, results revealed that individuals who highly valued self-transcendence displayed higher relationship quality (Table 1). This time results also revealed a positive effect for openness to change values. Results were the same when not controlling for relationship duration or age (Table S1 in the supplemental materials).

We used a bootstrapping method with PROCESS (Hayes & Preacher, 2013) with 5,000 iterations to test the indirect effects of pro-relational attitudes on the association between self-transcendence values and relationship quality. We did not control for the other values. Results revealed that the association between self-transcendence values and relationship quality was significantly but partially mediated by infidelity and sacrificing but not forgiving attitudes (Table 2). Because the association between openness values and relationship quality was not indicated in Studies 1 and 2, we did not explore it further through mediational analyses.

**Table 2.** Bootstrapping Analyses of Indirect Effects of Self-Transcendence Values and Relationship Quality Through Forgiving, Infidelity, and Sacrificing Attitudes (Study 3).

	Relationship quality		
	β	*SE*	95% CI
Total effect	0.35*	0.08	[0.31, 0.64]
Direct effect	0.14*	0.09	[0.02, 0.37]
Indirect effect forgiving attitudes	−0.04	0.03	[−0.11, 0.02]
Indirect effect infidelity attitudes	0.06*	0.03	[0.01, 0.11]
Indirect effect sacrificing attitudes	0.19*	0.05	[0.10, 0.30]

*Note.* Bootstrap sample size = 5,000 bootstrap samples; CI = 95% confidence interval.

* *p* < .05, *N* = 232.

### Discussion

Study 3 replicated and extended the results of Studies 1 and 2. People who attach higher importance to self-transcendence values exhibit higher relationship quality. Moreover, the findings revealed that attitudes toward (in)fidelity and sacrifice significantly mediated this association. Hence, an important initial conclusion, based on the first three studies, is that the extent to which people prioritize self-transcendence values is important for predicting romantic relationship quality because it is associated with more positive attitudes to pro-relationship behaviors (which putatively underlie relationship functioning).

## Study 4

In Study 4 we had two aims. First, using a pre-registered design, we aimed to examine the robustness of the association between self-transcendence and relationship quality through a comparison of value instruments in the same sample. Second, we further examined in what ways people’s values might be related to their romantic relationship quality. Like Study 3, Study 4 also followed value-attitude models (Boer & Fischer, 2013; Maio & Olson, 1995), construing values as guiding principles that shape value-expressive attitudes. Although the findings of Study 3 indicated that pro-relational attitudes underly this association, there are different ways in which these attitudes might manifest in relationship contexts. In addition to the specific pro-relational behavioral aspects considered in Study 3 (e.g., infidelity and sacrifice), there are relevant motivational aspects of pro-relational attitudes. These attitudes should shape the way people construe their relationships, directing people toward a more communal or intrinsic motivational perspective. Study 4 focused on these related motivational processes.

We focused on three variables in particular: communal strength, the extent of intrinsic motivation to be in the relationship, and feelings of entitlement. Theoretically, the first two mediators are integral to pro-relational attitudes observed as mediators in Study 3. That is, more positive attitudes to relationship-maintaining behavior should subsume higher communal strength and higher intrinsic relationship motivation. First, people higher in communal strength are particularly motivated to be responsive to their partner’s needs, without expectations of direct reciprocation (Mills et al., 2004). Higher communal strength is associated with providing more help to friends and being more satisfied (and having partners who are more satisfied) with one’s romantic relationships (Mills et al., 2004). We therefore tested whether communal strength mediates the association between self-transcendence values and relationship quality. Second, relationships are the most satisfied and the most stable when they are upheld and pursued for intrinsic reasons (Blais et al., 1990). It could be that people who endorse self-transcendence values see being in a relationship as central to their self, facilitating pro-relational attitudes in which relationships are instruments for self-completion. We therefore tested whether intrinsic relationship goals mediate the association between values and relationship quality by asking participants about their reasons for being in a relationship (Blais et al., 1990). In contrast, the third potential mediator, entitlement, is theoretically antithetical to pro-relational attitudes. Individuals with a high sense of entitlement are more self-oriented, have lower levels of accommodation, empathy, and perspective-taking, and less respect for their partner (Campbell et al., 2004). Hence, we expected that higher self-transcendence values are associated with lower feelings of entitlement, which (negatively) mediate the association between self-transcendence values and relationship quality.

### Method

#### Participants and Design

We recruited 350 British adults in romantic relationships through Prolific Academic in exchange for £1.00. This number was sufficient to detect a small to medium effect (*r* = .20; α = .05, 80% power), including 10% dropout due to the online nature of the study. Forty-eight participants were excluded from the analyses because they failed an instructed-response attention check (Kung et al., 2018), and an additional two participants were excluded because they were together for less than a year. The final sample consisted of 310 participants (72 men; 238 women) between 18 and 70 years of age (*M* = 37.42, *SD* = 10.98). Most of the participants were Caucasian (95%), the rest were Black or African American (2%), Asian or Pacific Islander (3%), or Other (1%). Most participants either had a college or university degree (61%) or some college education (21%). All relationships had lasted for at least a year at the time of participation (*M* = 11.78 years, *SD* = 9.44, ranging from 1 to 50 years), and most were heterosexual (*N* = 281).

#### Procedure

This study was preregistered (https://aspredicted.org/t3mr3.pdf). The procedure was similar to Studies 1 and 3, with data collected using Qualtrics software. After giving informed consent, participants were instructed that they would be asked about their value priorities and relationship well-being. The order of all questionnaires and items (within questionnaires) was randomized.

#### Measures

##### Values

As in Studies 1 and 3, we again used separate gender-matched versions of the PVQ (Schwartz et al., 2012) to measure values. The higher-order value domains demonstrated high reliability (.85 < Cronbach’s α < .89).

This sample also received the SSVS-19 (Lindeman & Verkasalo, 2005) to measure values. Instructions were identical to those of the SSVS in Study 3. Participants rated the importance of each value by writing down a score between −4 (*any values extremely opposed to the principles that guide you*) and +4 (*a value of supreme importance as a guiding principle in your life; ordinarily, there are relatively few such values*) next to each value. Within the final sample, due to a coding error, we excluded the answers on the SSVS-19 from 22 participants who had given responses outside the scale (i.e., answered −8 or +10). These participants manifested no abnormal responding in the rest of the study. The higher order value domains demonstrated low to moderate reliability (.58 < Cronbach’s α < .68).

##### Relationship Quality

Identical to Studies 1 to 3, we included the PRQC (Fletcher et al., 2000) to measure relationship quality, showing excellent reliability (*M* = 5.88, *SD* = .96, Cronbach’s α = .96).

##### Mediators

We explored the mediating roles of communal strength, intrinsic relationship motivation, and entitlement.

##### Communal Strength

We measured communal strength with a 10-item questionnaire (Mills et al., 2004; *M* = 8.84, *SD* = 1.23, Cronbach’s α = .78). Participants were asked to indicate how far they would go to respond to their partner’s needs (e.g., “How far would you be willing to go visit your partner?”). Responses were given on an 11-point Likert-type scale ranging from 0 (*not at all*) to 10 (*extremely*).

##### Relationship Motivation

To assess intrinsic relationship motivation, participants were asked open-ended question: “Why are you in a romantic relationship with your partner? Please describe:.” The minimum required response length was set at 100 characters. Example answers were “I am in a romantic relationship with my partner for companionship, because I love her and I would do anything for her” and “Because we have been married for 15 years and have 4 children together. Romance is not everything in a relationship.” Four independent raters coded how much extrinsic vs. intrinsic motivation for being in the relationship was expressed from 1 (*extrinsically motivated*) to 7 (*intrinsically motivated*). Interrater reliability was high (α = .87; *M* = 5.62, *SD* = 1.16); hence, the ratings were averaged into one single score.

##### Entitlement

We presented participants with the Narcissistic Admiration and Rivalry Questionnaire (NARQ, Back et al., 2013); *M* = 2.44, *SD* = 0.68, Cronbach’s α = .88. The NARQ asks participants to indicate how much they agreed with each of 18 statements (e.g., “I secretly take pleasure in the failure of my rivals”). Responses were given on a 6-point Likert-type scale ranging from 1 (*I do not agree at all*) to 6 (*I agree completely*). We used the mean score of all 18 items as an indicator of entitlement.

#### Statistical Analysis

The analytic strategy slightly deviated from our pre-registered plan. Instead of conducting (partial) correlation analyses to examine associations between values and relationship quality (see Table S3 in the supplemental materials), we conducted multiple regression analyses. This improvement enabled us to examine which association(s) prevails over others while maintaining a consistent analytical approach across studies.

### Results

Replicating previous results, we found a uniquely positive effect for self-transcendence values on relationship quality, regardless of whether values were measured with the PVQ or the SSVS. We did not find effects for self-enhancement, openness to change, or conservation values. Results remained identical when not controlling for relationship duration or age (Table S1 in the supplemental materials).

#### Analyses of Potential Mediators

To test the indirect effects, we used a bootstrapping method with PROCESS (Hayes & Preacher, 2013). We ran 5,000 iterations for a model with self-transcendence values as measured with the PVQ and for a model with self-transcendence values as measured with the SSVS. We did not control for the other values. Results revealed that associations between self-transcendence values and relationship quality were significantly and fully mediated by communal strength and intrinsic relationship motivation, regardless of how the values were measured (Table 3). There was no reliable evidence of mediation via entitlement.

**Table 3.** Bootstrapping Analyses of Indirect Effects of Self-Transcendence Values (Measured With the PVQ or SSVS) and Relationship Quality Through Communal Strength, Intrinsic Relationship Motivation, and Entitlement (Study 4).

	PVQ			SSVS		
	β	*SE*	95% CI	β	*SE*	95% CI
Total effect	0.29*	0.08	[0.27, 0.58]	0.27*	0.06	[0.15, 0.37]
Direct effect	0.09	0.07	[−0.01, 0.27]	0.08	0.05	[−0.03, 0.18]
Indirect effect communal strength	0.12*	0.03	[0.07, 0.20]	0.10*	0.03	[0.05, 0.16]
Indirect effect relationship motivation	0.08*	0.03	[0.03, 0.13]	0.09*	0.03	[0.03, 0.16]
Indirect effect entitlement	−0.002	0.01	[−0.01, 0.01]	−0.005	0.004	[−0.01, 0.01]

*Note*: Bootstrap sample size = 5,000 bootstrap samples; PVQ = Portrait Values Questionnaire; SSVS = Short Schwartz Value Survey; CI = 95% confidence interval.

* *p* < .05, *N* = 310 (PVQ), *N* = 288 (SSVS).

### Discussion

The findings of Study 4 reveal that people who more strongly endorse self-transcendence values exhibit higher relationship quality. This association is statistically mediated by enhanced levels of communal strength and intrinsic relationship motivation. However, we found no strong evidence for the role of entitlement. It was unrelated to values, and it was also unrelated to relationship quality (controlling for communal orientation and intrinsic motivation). While the explanation for this lack of effect is difficult to pinpoint, one reason may be that extreme levels of self-reported entitlement were rare in the population we studied. Overall, these findings reveal two important relationship variables through which stronger self-transcendence values predict higher relationship quality: communal strength and intrinsic relationship motivation.

## Study 5

The results from Study 4 again found that prioritizing self-transcendence values predicts higher relationship quality while revealing mediation of this association through communal strength and intrinsic motivation. An interesting feature of these mediators is that they have similar implications for the person holding the values (i.e., the actor), while the mediator’s effect on the relationship quality of the other person in the dyadic relationship is less clear (i.e., the partner). Specifically, the effect of self-transcendence through these mediators implies a transactional effect of the actor’s values on their partner’s relationship quality. After all, people high in communal strength are more likely to forgo their own self-interest in conflict situations in favor of their partner or their relationship (Day et al., 2015). Furthermore, high intrinsic relationship motivation might also translate into more prosocial behavior toward the partner, in an effort to sustain and enhance the treasured relationship (Blais et al., 1990). Consequently, both mediators might enhance the perceptions of relationship quality for both actor and partner through the actor’s positive behavior.

However, the found mediational effects are not by themselves evidence for a behavioral mechanism connecting self-transcendence values to relationship quality. For example, it is possible that partners’ relationship quality is not sensitive to the changes in relationship quality that are affecting the actor. Or that self-transcendence values indeed change behaviors, but only when personally significant to the partner. Moreover, it is thinkable that high intrinsic relationship motivation stems from an actor defining their identity as being in a relationship (i.e., relational-interdependent self-construal; Cross et al., 2000), which has been associated with positive illusions of relationship quality that would not affect the relationship perception of a partner (Boucher, 2014). The mediational role of pro-relational attitudes observed in Study 3 is also not unequivocally clear about a behavioral impact on relationships. Although these attitudes should predict behavior in a relationship, in line with abundant evidence for attitude-behavior linkages (Armitage & Conner, 2001; Glasman & Albarracín, 2006), the effect may have more to do with partners’ motivations to perform the pro-relational behaviors than the behaviors per se.

To test whether the effects are related to the partner’s detection of positive actions toward them, we analyzed responses from a dyadic data set in Study 5. The data set for this study was initially collected for another purpose. However, it contained all the necessary measurements for us to test our hypotheses. We expected to replicate our previous effects, showing that an actor’s higher self-transcendence values relate to the actor reporting better relationship quality. In addition, we expected an actor’s self-transcendence values to relate to their partner’s relationship quality.

### Method

#### Participants

We recruited 568 romantic couples for a study on the COVID-19 pandemic, personality, and romantic relationships through prolific in exchange for £1.66 per person. We excluded six respondents who failed an instructed-response attention check (Kung et al., 2018) and their partners. The remaining 562 couples (1,124 individuals, 542 men, 580 women, 1 non-binary, 1 other gender, average relationship duration = 116.38 months, *SD* = 98.84 months) comprised individuals aged between 18 and 82 years (*M* = 34.05, *SD* = 9.99). Most couples in this sample were heterosexual (92%) and cohabiting (88%), but only a minority were married (46%).

#### Procedure

Individual respondents received information about the study and were instructed to invite their partners to participate through the recruitment portal (Prolific Academic). After informed consent from both partners had been obtained, they were invited to complete the main questionnaire. All instruments were presented in two blocks, a personality block (i.e., values and personality traits) and an outcome block (i.e., relationship quality and COVID-19-related questions). The order of these blocks was randomized. The order of the individual questionnaires within the blocks was also randomized, just like the order of all items in the value, trait, and relationship quality instruments.

#### Measures

##### Values

Respondents completed the SSVS as reported in Study 2. The higher order value domains demonstrated moderate to good reliability (.64 < Cronbach’s α < .74).

##### Relationship Quality

Respondents answered a six-item short version of the PRQC on a scale from 1 (*not at all*) to 7 (*extremely*). Following Fletcher et al.’s (2000) recommendations, the short scale only contained one item for each of the PRQC’s six facets of relationship quality (satisfaction, commitment, intimacy, trust, passion, and love). We chose the item most directly measuring the facet in question (e.g., “How satisfied are you with your relationship,” “How committed are you to your relationship”). This short scale had very good internal consistency (α = .88, *M* = 6.24, *SD* = 0.82).

##### Other Scales

Respondents also completed 30 items measuring the big five personality traits (the XS5; Konstabel et al., 2017), 10 items asking about self and partner-perceived adherence to infection prevention behaviors (Blagov, 2020), 14 items measuring moralization of infection prevention behaviors (Feinberg et al., 2019), and five items about COVID-related relationship conflict (adapted from Spanier, 1976). Because these measurements are not the focus of the current investigation, their results will not be further reported in this article.

#### Statistical Analysis

To analyze the dyadic effects of values on relationship quality, while accounting for the inherent lack of independence present in dyadic data, we applied the Actor Partner Interdependence Model using Multilevel Modeling (APIM; Cook & Kenny, 2005). As our sample included same-sex couples, we chose to treat partners as indistinguishable (i.e., as actors and partners instead of—for example—wives and husbands) for this analysis. To obtain standardized coefficients, we z-standardized all variables before the analysis. As before, we controlled for relationship duration in all analyses.

### Results

Table 4 shows APIM results for all four higher-order value types. As before, we found that actors who highly value self-transcendence report higher relationship quality (β(actor) = 0.12, *p* < .001). However, against our predictions, the actor’s self-transcendence values did not relate to the partner’s relationship quality (β(partner) = 0.02, *p* = .533). Interestingly, a similar pattern was evident for conservation values, where actors who highly value conservation report higher relationship quality (β(actor) = 0.08, *p* = .005), while their own conservation values did not relate to the partner’s relationship quality (β(partner) = −0.03, *p* = .266). Nonetheless, we do not further consider the effect of conservation values on the actor’s own relationship quality because this association was not evident in our other studies. No significant actor or partner effects were found for openness or self-enhancement values.

**Table 4.** APIM Model Results for All Values Predicting the Actor’s Relationship Quality (Study 5).

	Self-transcendence	Self-enhancement	Openness to change	Conservation
Standardized regression coefficients				
β(actor)	0.12***	0.01	0.02	0.06**
β(partner)	0.02	0.00	0.01	−0.03
Relationship duration	−0.00	−0.00	−0.00	−0.00
Age				
β(actor)	0.00	0.00	0.00	0.00
β(partner)	−0.01*	−0.01	−0.01	−0.01*
*R* ^2^	.03***	.01	.01	.01*

*Note.* The *R*^2^ values were computed using Edwards et al.’s (2008) method for calculating the variance explained by all fixed effects in a multilevel model.

* *p* < .05. **p < .01. ***p < .001.

### Discussion

The results of Study 5 were in line with our previous findings that placing a higher value on self-transcendence was associated with higher relationship quality. However, contrary to our expectations based on the interpersonal mechanism associated with communal strength, we did not find that a partner’s self-transcendence values were associated with their significant other’s relationship quality. Perhaps a partner perceives a real change in the communal nature of the relationship, but this does not reach a sufficient threshold for the significant other to notice. Another explanation might be that, as mentioned, the link between values and relationship quality is more strongly driven by the actor’s intrinsic relationship motivation, possibly resulting in beneficial illusions about the quality of their relationship.

## Meta-Analytic Summary

A meta-analysis was performed for the correlations between self-transcendence values and relationship quality. As recommended by Goh et al. (2016), we used fixed effects in which the mean effect size (i.e., mean correlation) was weighted by sample size. All correlations were Fisher’s z transformed for analyses and converted back to Pearson correlations for presentation. Across the four studies (and five correlations), both uncentered and centered self-transcendence values were significantly positively associated with relationship quality (see Table 5).

**Table 5.** Meta-Analytic Results of Correlations Between Uncentered and Centered Self-Transcendence Values and Relationship Quality.

	*N*	Uncentered	Centered
Study 1—PVQ	237	.19	.08
Study 2—SSVS	222	.20	.13
Study 3—PVQ	233	.35	.19
Study 4—PVQ	310	.28	.17
Study 4—SSVS	288	.24	.12
Study 5—SSVS	1,024	.12	.07
*M r_z_*		.29	.22
*M r*		.28	.22
Combined *Z*		9.83***	5.45***

*Note. M rz* = weighted mean correlation (Fisher’s *z* transformed). *M r* = weighted mean correlation (converted from *r_z_* to *r*). PVQ = Portrait Values Questionnaire; SSVS = Short Schwartz Value Survey.

*** *p* < .001.

## General Discussion

The present research examined the association between people’s personal values and romantic relationship quality. Across five studies, using multiple online samples with a relatively broad age range, different value instruments, among individuals and couples, we found that the endorsement of self-transcendence values was strongly and consistently associated with enhanced romantic relationship quality, whereas the endorsement of self-enhancement, openness to change, and conservation values was weakly and inconsistently associated with romantic relationship quality. In addition, we found that the association between self-transcendence values and romantic relationship quality can be explained by enhanced levels of pro-relational attitudes and by two relevant motivational variables that support relationships: communal strength and intrinsic relationship motivation.

Our findings have important implications for both the study of romantic relationships and research on human values. Advancing our knowledge about romantic relationships, we present robust evidence of the influence of valuing self-transcendence. We find that holding certain prosocial ideals to be important can influence the way in which people perceive their own interpersonal relationships. Extending our knowledge about human values, we show evidence for interesting mechanisms by which they influence relationship quality. Prior to the current work, the vast majority of research on human values has occurred outside of the context of romantic relationships, although values are often regarded as being core to relationship harmony (Lee et al., 2009; Leikas et al., 2018; Sprecher & Regan, 2002; Watson et al., 2004). Our research shows that, in a relationship context, the focus on helping others conveyed by self-transcendence values manifests as more pro-relational attitudes, more intrinsic motivation to maintain relationships, and higher levels of responsiveness to their partner’s needs.

The value types arrayed on the second dimension of the value structure, openness to change versus conservation, were less relevant for understanding romantic relationship functioning, exhibiting only weak and inconsistent correlations with relationship quality. This finding is interesting in light of evidence that paying less attention to attractive alternatives (Simpson & Gangestad, 1990) is related to higher relationship quality, along with evidence that the prevention of relationship boredom is crucial in romantic relationships (Tsapelas et al., 2009). Our data reveal no consistent role for either set of values. This raises interesting questions for future research. It may be the case that the roles of these values are nuanced. For example, openness values may be relatively important early in relationships (to facilitate intimacy and fun), and conservation values may grow in importance later in relationships (to preserve them as relationship excitement decreases). This speculation requires direct testing for a more complete understanding of the role of this values dimension.

More relevant here, Studies 3 and 4 revealed a mechanism through which self-transcendence values predict relationship quality. These values were positively associated with relationship quality through enhanced pro-relational attitudes, communal strength, and intrinsic relationship motivation. The results of Study 5 showed that a person’s self-transcendence values did not predict their partner’s view of the relationship. This is in line with a meta-analysis by Joel et al. (2020) demonstrating little evidence for partner effects in promoting relationship quality. Although speculating, it could also be that this lack of a partner effect indicates that individuals in a romantic relationship do not pick up their partners’ self-transcendence value preferences or perhaps only when reaching a certain threshold. Alternatively, it could be that the endorsement of self-transcendence values moves relationships to become more central in a person’s life and self, which in turn makes being in the relationship an instrument for the person’s self-completion (i.e., intrinsic relationship motivation—explanation).

Before concluding, it is important to note several limitations of the present research and to make suggestions for future research. First, although the meta-analytic effects underscore the consistency of the findings, the effect sizes are relatively small. Second, the reliabilities of the higher order value domains were weak or only moderate when measured with the SSVS in Study 4. This low reliability might be explained by the answering format (−4 to 4) we employed in Study 4, which produced worse internal consistency than the −1 to 7 answering format in Study 2. Third, the cross-sectional design of the present studies prevented us from drawing conclusions about the causal effects of value priorities on romantic relationship quality. Supporting the notion that personal values are the cause of relationship quality, values have been shown to be resistant to change, with high rank-order stability over time (Vecchione et al., 2016). Contrary to this notion, however, there is evidence for values being susceptible to change due to environmental influences to some degree (Bardi & Goodwin, 2011). Longitudinal and experimental research could help to uncover such causal mechanisms. Finally, future research could extend the investigation to lower-order value types. Specifically, the distinction between universalism (welfare for wider society and nature) and benevolence (benefiting the individuals in the immediate environment) could be of interest for further understanding how and when self-transcendence values relate to relationship quality. In supplement Table 6 the correlations between the lower-order value types as measured with the PVQ and relationship quality can be found. These findings indeed suggest that some specific values may be more strongly associated with relationship quality than others. Unfortunately, however, the short-form SSVS measures we have used do not allow us to reliably assess lower-order values, so this possibility requires replication before drawing firm conclusions.

In conclusion, our findings emphasize the importance of understanding the role of values in romantic relationship functioning. By showing that people who more strongly endorse benevolent, self-transcendent values report better quality relationships, the findings may ultimately even help to explain why some relationships break down where others prosper. We hope that the current findings offer a springboard to further explore the role of values in romantic relationship functioning and well-being.

## Supplemental Material

sj-docx-1-psp-10.1177_01461672231156975 – Supplemental material for Values in Romantic Relationships Supplemental material, sj-docx-1-psp-10.1177_01461672231156975 for Values in Romantic Relationships by Reine C. van der Wal, Lukas F. Litzellachner, Johan C. Karremans, Nadia Buiter, Jamie Breukel and Gregory R. Maio in Personality and Social Psychology Bulletin

## Notes

1. We expected values to contribute to relationship quality to prosocial interpersonal action and motivation. As such, assessing higher-order values (i.e., self-transcendence, self-enhancement), rather than lower-order values (i.e., universalism, power) would provide more reliable measurements of these broad motivational tendencies.
