# Supplementary material for: Values in Romantic Relationships
Source: Pers Soc Psychol Bull. 2023 Mar 21;50(7):1066–79. doi: 10.1177/01461672231156975 (PMC11143760; doi:10.1177/01461672231156975)
Supplement: sj-docx-1-psp-10.1177_01461672231156975 – Supplemental material for Values in Romantic Relationships [file sj-docx-1-psp-10.1177_01461672231156975.docx]

**Supplementing Information for**

**Values in Romantic Relationships**

|  | **Study 1: Relationship Quality by PVQ** | | | | |  | **Study 2: Relationship Quality by SSVS** | | | | |  | **Study 3: Relationship Quality by PVQ** | | | | |  |
| --- | --- | --- | --- | --- | --- | --- | --- | --- | --- | --- | --- | --- | --- | --- | --- | --- | --- | --- |
|  | *β* | CI_low_ | CI_high_ | *t* | *p* |  | *β* | CI_low_ | CI_high_ | *t* | *p* |  | *β* | CI_low_ | CI_high_ | *t* | *p* |  |
| Self-Transcendence | 0.18 | 0.03 | 0.37 | 2.27 | .024 |  | 0.22 | 0.02 | 0.45 | 2.13 | .004 |  | 0.17 | 0.01 | 0.33 | 2.09 | .038 |  |
| Self-Enhancement | -0.03 | -0.19 | 0.12 | -0.48 | .630 |  | -0.13 | -0.23 | 0.02 | -1.68 | .110 |  | -0.12 | -0.25 | 0.02 | -1.73 | .084 |  |
| Openness to Change | -0.03 | -0.21 | 0.14 | -0.40 | .692 |  | 0.01 | -0.17 | 0.15 | 3.64 | .870 |  | 0.28 | 0.13 | 0.43 | 3.64 | <.001 |  |
| Conservation | 0.11 | -0.02 | 0.28 | 1.68 | .095 |  | 0.11 | -0.07 | 0.27 | 1.16 | .180 |  | 0.09 | -0.05 | 0.23 | 1.28 | .203 |  |
|  | *F*(4, 232) = 2.92, *p* = .022, *R^2^* = .05 | | | | |  | *F*(4, 226) = 10.15, *p* < .001, *R^2^* = .19 | | | | |  | *F*(4, 228) = 11.66, *p* < .001, *R^2^* = .17 | | | | |  |
|  | **Study 4: Relationship Quality by PVQ** | | | | |  | **Study 4: Relationship Quality by SSVS** | | | | |  | **Study 5: Relationship Quality by SSVS** | | | | | |
|  | *β* | CI_low_ | CI_high_ | *t* | *p* |  | *β* | CI_low_ | CI_high_ | *t* | *p* |  | *β* | CI_low_ | CI_high_ | *t* | *p* | |
| Self-Transcendence | 0.21 | 0.07 | 0.35 | 2.91 | .004 |  | 0.22 | 0.08 | 0.33 | 3.29 | .001 |  | 0.10 | 0.04 | 0.16 | 3.09 | .002 | |
| Self-Enhancement | -0.08 | -0.20 | 0.04 | -1.30 | .196 |  | 0.04 | -0.07 | 0.15 | 0.73 | .467 |  | 0.01 | -0.05 | 0.07 | 0.50 | .618 | |
| Openness to Change | 0.08 | -0.07 | 0.21 | 1.02 | .308 |  | 0.00 | -0.11 | 0.12 | 0.03 | .975 |  | -0.02 | -0.08 | 0.04 | -0.84 | .400 | |
| Conservation | 0.08 | -0.05 | 0.19 | 1.20 | .232 |  | 0.08 | -0.04 | 0.19 | 1.29 | .197 |  | 0.03 | -0.03 | 0.09 | 1.13 | .260 | |
|  | *F*(4, 305) = 6.99, *p* < .001, *R^2^* = .08 | | | | |  | *F*(4, 283) = 4.99, *p* = .001, *R^2^* = .07 | | | | |  | *F*(4, 557) = 4.20, *p* < .001, *R^2^* = .01 | | | | | |
| *Note.* Coefficients from Study 5 represent fixed effects in a multilevel regression model with random intercepts for each couple. The R^2^-values were computed using Edwards et al.'s (2008) method for calculating the variance explained by all fixed effects in a multilevel model. | | | | | | | | | | | | | | | | | | |

**Table S1**
*Regression of Relationship Quality with Uncentered Value Priorities Excluding Relationship Duration and Age* (Study 1-5)

| **Table S2** *Regression of Relationship Quality with Uncentered Value Priorities Including Relationship Duration and Age* (Study 1-5) | | | | | | | | | | | | | | | | | |  |
| --- | --- | --- | --- | --- | --- | --- | --- | --- | --- | --- | --- | --- | --- | --- | --- | --- | --- | --- |
|  | **Study 1: Relationship Quality by PVQ** | | | | |  | **Study 2: Relationship Quality by SSVS** | | | | |  | **Study 3: Relationship Quality by PVQ** | | | | |  |
|  | *β* | CI_low_ | CI_high_ | *t* | *p* |  | *β* | CI_low_ | CI_high_ | *t* | *p* |  | *β* | CI_low_ | CI_high_ | *t* | *p* |  |
| Self-Transcendence | 0.16 | 0.01 | 0.36 | 2.10 | .037 |  | 0.25 | 0.07 | 0.42 | 2.75 | .007 |  | 0.18 | 0.02 | 0.34 | 2.19 | .029 |  |
| Self-Enhancement | -0.12 | -0.31 | 0.03 | -1.61 | .108 |  | 0.03 | -0.12 | 0.17 | 0.38 | .702 |  | -0.11 | -0.25 | 0.03 | -1.62 | .108 |  |
| Openness to Change | 0.03 | -0.14 | 0.21 | 0.41 | .680 |  | -0.08 | -0.24 | 0.08 | -0.98 | .331 |  | 0.29 | 0.14 | 0.44 | 3.85 | <.001 |  |
| Conservation | 0.12 | -0.02 | 0.30 | 1.72 | .087 |  | -0.01 | -0.18 | 0.16 | -0.11 | .912 |  | 0.06 | -0.08 | 0.21 | 0.83 | .407 |  |
| Relationship Duration | 0.18 | -0.001 | 0.41 | 1.96 | .051 |  | -0.00 | -0.00 | 0.00 | -0.61 | .546 |  | 0.03 | -0.11 | 0.17 | 0.44 | .661 |  |
| Age | -0.30 | -0.55 | -0.12 | -3.11 | .002 |  | -0.39 | -0.60 | -0.18 | -3.66 | <.001 |  | 0.04 | -0.10 | 0.18 | 0.54 | .592 |  |
|  | *F*(6, 228) = 3.47, *p* = .003, *R^2^* = .08 | | | | |  | *F*(6, 216) = 2.08, *p* = .070, *R^2^* = .02 | | | | |  | *F*(6, 225) = 8.18, *p* < .001, *R^2^* = .18 | | | | |  |
|  | **Study 4: Relationship Quality by PVQ** | | | | |  | **Study 4: Relationship Quality by SSVS** | | | | |  | **Study 5: Relationship Quality by SSVS** | | | | | |
|  | *β* | CI_low_ | CI_high_ | *t* | *p* |  | *β* | CI_low_ | CI_high_ | *t* | *p* |  | *β* | CI_low_ | CI_high_ | *t* | *p* | |
| Self-Transcendence | 0.22 | 0.07 | 0.35 | 3.01 | .003 |  | 0.23 | 0.09 | 0.34 | 3.46 | .001 |  | 0.10 | 0.04 | 0.16 | 3.17 | .002 | |
| Self-Enhancement | -0.13 | -0.26 | 0.01 | -1.90 | .058 |  | 0.02 | -0.10 | 0.13 | 0.26 | .797 |  | 0.01 | -0.05 | 0.07 | 0.45 | .651 | |
| Openness to Change | 0.08 | -0.06 | 0.22 | 1.07 | .287 |  | 0.03 | -0.10 | 0.14 | 0.44 | .658 |  | -0.01 | -0.07 | 0.05 | -0.38 | .704 | |
| Conservation | 0.09 | -0.04 | 0.21 | 1.32 | .188 |  | 0.08 | -0.05 | 0.19 | 1.16 | .245 |  | 0.01 | -0.05 | 0.07 | 1.13 | .648 | |
| Relationship Duration | 0.05 | -0.11 | 0.20 | 0.62 | .535 |  | 0.04 | -0.12 | 0.20 | 0.52 | .600 |  | -0.00 | -0.00 | 0.00 | -2.31 | .022 | |
| Age | -0.18 | -0.32 | -0.02 | -2.21 | .028 |  | -0.17 | -0.32 | -0.01 | -2.07 | .039 |  | -0.06 | -0.17 | 0.05 | -1.48 | .140 | |
|  | *F*(6, 303) = 5.78, *p* < .001, *R^2^* = .10 | | | | |  | *F*(6, 281) = 4.25, *p* < .001, *R^2^* = .08 | | | | |  | *F*(6, 557) = 4.20, *p* < .001, *R^2^* = .01 | | | | | |
| *Note.* Including values that overlap multiple higher order domains (Humility and Face added to Conservation values, Hedonism added to Openness values). | | | | | | | | | | | | | | | | | | |

| **Table S3** *Descriptive Statistics, Reliabilities, and Zero-order Correlations for all Uncentered Values and Relationship Quality* (Study 1-5) | | | | | | | | | | | | | | | | | |
| --- | --- | --- | --- | --- | --- | --- | --- | --- | --- | --- | --- | --- | --- | --- | --- | --- | --- |
|  | **Study 1: Relationship Quality by PVQ** | | | | |  | **Study 2: Relationship Quality by SSVS** | | | | |  | **Study 3: Relationship Quality by PVQ** | | | | |
|  | SET | SEN | OTC | CON | RQ |  | SET | SEN | OTC | CON | RQ |  | SET | SEN | OTC | CON | RQ |
| *M* (*SD*) | 4.74 (0.60) | 3.45 (1.08) | 4.71 (0.62) | 4.22 (0.80) | 5.80 (1.12) |  | 6.92 (1.16) | 4.74 (1.71) | 7.05 (1.13) | 6.32 (1.13) | 5.69 (1.06) |  | 4.73 (0.74) | 3.23 (1.06) | 4.63 (0.74) | 4.14 (0.86) | 5.93 (0.99) |
| Reliability | .82 | .90 | .82 | .88 | .95 |  | .81 | .81 | .71 | .76 | .98 |  | .91 | .89 | .88 | .88 | .95 |
| Self-Transcendence | - | .02 | .49*** | .31*** | .19* |  | - | .06 | .56*** | .50*** | .20** |  | - | .04 | .50*** | .46*** | .35*** |
| Self-Enhancement | - | - | .36*** | .24*** | -.01 |  | - | - | .13^t^ | .31*** | .03 |  | - | - | .30*** | .26*** | -.01 |
| Openness to Change | - | - | - | .22* | .11^t^ |  | - | - | - | .33*** | .02 |  | - | - | - | .12^t^ | .35*** |
| Conservation | - | - | - | - | .13^t^ |  | - | - | - | - | .12^t^ |  | - | - | - | - | .16* |
|  | **Study 4: Relationship Quality by PVQ** | | | | |  | **Study 4: Relationship Quality by SSVS** | | | | |  | **Study 5: Relationship Quality by SSVS** | | | | |
|  | SET | SEN | OTC | CON | RQ |  | SET | SEN | OTC | CON | RQ |  | SET | SEN | OTC | CON | RQ |
| *M* (*SD*) | 4.68 (0.66) | 3.31 (0.87) | 4.46 (0.70) | 4.02 (0.73) | 5.88 (0.96) |  | 2.42 (0.96) | -0.31 (1.51) | 2.40 (0.97) | 1.84 (1.04) | 5.88 (0.96) |  | 5.05 (1.19) | 2.23 (1.65) | 4.92 (1.72) | 4.47 (1.17) | 6.24 (0.82) |
| Omega | .87 | .88 | .85 | .85 | .96 |  | .68 | .72 | .54 | .61 | .96 |  | .79 | .74 | .67 | .70 | .93 |
| Self-Transcendence | - | .05 | .52*** | .39*** | .28*** |  | - | -.04 | .34*** | .35*** | .24*** |  | - | -.04 | .35*** | .40*** | .12*** |
| Self-Enhancement | - | - | .39*** | .34*** | -.03 |  | - | - | .17** | .24*** | .05 |  | - | - | .25*** | .26*** | .02 |
| Openness to Change | - | - | - | .16** | .16** |  | - | - | - | .09 | .12^t^ |  | - | - | - | .23*** | .03 |
| Conservation | - | - | - | - | .14* |  | - | - | - | - | .14* |  | - | - | - | - | .05^t^ |
| *Note.* Including values that overlap multiple higher order domains (Humility and Face added to Conservation values, Hedonism added to Openness values). SET = Self-Transcendence, SEN = Self-Enhancement, OTC = Openness to Change, CON = Conservation, RQ = Relationship Quality. Reliability shows McDonald’s ω.  ^t^*p* < .1, **p* < .05, ***p* < .01, ****p* < .001 | | | | | | | | | | | | | | | | | |

| **Table S4** *Descriptive Statistics, Reliabilities, and Zero-order Correlations for all Centered Values and Relationship Quality* (Study 1-5) | | | | | | | | | | | | | | | | | | |
| --- | --- | --- | --- | --- | --- | --- | --- | --- | --- | --- | --- | --- | --- | --- | --- | --- | --- | --- |
|  | **Study 1: Relationship Quality by PVQ** | | | | | |  | **Study 2: Relationship Quality by SSVS** | | | | |  | **Study 3: Relationship Quality by PVQ** | | | | |
|  | SET | SEN | | OTC | CON | RQ |  | SET | SEN | OTC | CON | RQ |  | SET | SEN | OTC | CON | RQ |
| *M* (*SD*) | 4.74 (0.60) | 3.45 (1.08) | | 4.71 (0.62) | 4.22 (0.80) | 5.80 (1.12) |  | 6.92 (1.16) | 4.74 (1.71) | 7.05 (1.13) | 6.32 (1.13) | 5.69 (1.06) |  | 4.73 (0.74) | 3.23 (1.06) | 4.63 (0.74) | 4.14 (0.86) | 5.93 (0.99) |
| Reliability | .82 | .90 | | .82 | .88 | .95 |  | .81 | .81 | .71 | .76 | .98 |  | .91 | .89 | .88 | .88 | .95 |
| Self-Transcendence | - | -.57*** | | .14* | -.37*** | .08 |  | - | -.64*** | .16* | -.22*** | .13* |  | - | -.64*** | .08 | -.29*** | .19* |
| Self-Enhancement | - | - | | -.10 | -.36*** | -.10 |  | - | - | -.36*** | -.25*** | -.06 |  | - | - | -.07 | -.27*** | -.21** |
| Openness to Change | - | - | | - | -.63*** | -.01 |  | - | - | - | -.38*** | -.12 |  | - | - | - | -.74*** | .16* |
| Conservation | - | - | | - | - | .03 |  | - | - | - | - | .00 |  | - | - | - | - | -.10 |
|  | **Study 4: Relationship Quality by PVQ** | | | | | |  | **Study 4: Relationship Quality by SSVS** | | | | |  | **Study 5: Relationship Quality by SSVS** | | | | |
|  | SET | SEN | OTC | | CON | RQ |  | SET | SEN | OTC | CON | RQ |  | SET | SEN | OTC | CON | RQ |
| *M* (*SD*) | 4.68 (0.66) | 3.31 (0.87) | 4.46 (0.70) | | 4.02 (0.73) | 5.88 (0.96) |  | 2.42 (0.96) | -0.31 (1.51) | 2.40 (0.97) | 1.84 (1.04) | 5.88 (0.96) |  | 5.05 (1.19) | 2.23 (1.65) | 4.92 (1.72) | 4.47 (1.17) | 6.24 (0.82) |
| Omega | .87 | .88 | .85 | | .85 | .96 |  | .68 | .72 | .54 | .61 | .96 |  | .79 | .74 | .67 | .70 | .93 |
| Self-Transcendence | - | -.67*** | .10 ^t^ | | -.31*** | .17** |  | - | -.59*** | -.03 | -.23*** | .12* |  | - | -.55*** | .07* | .06* | .07* |
| Self-Enhancement | - | - | -.04 | | -.25*** | -.18** |  | - | - | -.18** | -.11^t^ | -.06 |  | - | - | -.28*** | -.37*** | -.03 |
| Openness to Change | - | - | - | | -.75*** | .03 |  | - | - | - | -.50*** | -.03 |  | - | - | - | -.43*** | -.04 |
| Conservation | - | - | - | | - | -.01 |  | - | - | - | - | -.02 |  | - | - | - | - | -.01 |
| *Note.* Including values that overlap multiple higher order domains (Humility and Face added to Conservation values, Hedonism added to Openness values). SET = Self-Transcendence, SEN = Self-Enhancement, OTC = Openness to Change, CON = Conservation, RQ = Relationship Quality. Reliability shows McDonald’s ω.  ^t^*p* < .1, **p* < .05, ***p* < .01, ****p* < .001 | | | | | | | | | | | | | | | | | | |

| **Table S5** *Regression of Relationship Quality with Centered Self-transcendence Value Priorities Including Relationship Duration and Age* (Study 1-5) | | | | | | | | | | | | | | | | | |  |
| --- | --- | --- | --- | --- | --- | --- | --- | --- | --- | --- | --- | --- | --- | --- | --- | --- | --- | --- |
|  | **Study 1: Relationship Quality by PVQ** | | | | |  | **Study 2: Relationship Quality by SSVS** | | | | |  | **Study 3: Relationship Quality by PVQ** | | | | |  |
|  | *β* | CI_low_ | CI_high_ | *t* | *p* |  | *β* | CI_low_ | CI_high_ | *t* | *p* |  | *β* | CI_low_ | CI_high_ | *t* | *p* |  |
| Self-Transcendence | 0.11 | -0.02 | 0.27 | 1.72 | .086 |  | 0.18 | 0.02 | 0.35 | 2.15 | .032 |  | 0.19 | 0.06 | 0.32 | 2.90 | .004 |  |
| Relationship Duration | 0.20 | 0.01 | 0.43 | 2.08 | .038 |  | 0.00 | 0.00 | 0.00 | 2.15 | .033 |  | 0.04 | -0.10 | 0.19 | 0.58 | .562 |  |
| Age | -0.26 | -0.50 | -0.07 | -2.66 | .008 |  | -0.03 | -0.05 | -0.01 | -3.66 | .001 |  | -0.02 | -0.15 | 0.15 | -0.03 | .980 |  |
|  | *F*(3, 231) = 2.81, *p* = .040, *R^2^* = .04 | | | | |  | *F*(3, 218) = 5.04, *p* = .002, *R^2^* = .05 | | | | |  | *F*(3, 228) = 3.01, *p* = .031, *R^2^* = .04 | | | | |  |
|  | **Study 4: Relationship Quality by PVQ** | | | | |  | **Study 4: Relationship Quality by SSVS** | | | | |  | **Study 5: Relationship Quality by SSVS** | | | | | |
|  | *β* | CI_low_ | CI_high_ | *t* | *p* |  | *β* | CI_low_ | CI_high_ | *t* | *p* |  | *β* | CI_low_ | CI_high_ | *t* | *p* | |
| Self-Transcendence | 0.20 | 0.08 | 0.30 | 3.47 | <.001 |  | 0.13 | 0.02 | 0.24 | 2.26 | .025 |  | 0.05 | 0.01 | 0.10 | 2.25 | .026 | |
| Relationship Duration | 0.06 | -0.10 | 0.21 | 0.74 | .460 |  | 0.03 | -0.13 | 0.19 | 0.37 | .712 |  | -0.00 | -0.01 | 0.00 | -2.60 | .009 | |
| Age | -0.16 | -0.31 | -0.003 | -2.00 | .046 |  | -0.13 | -0.29 | 0.03 | -1.57 | .118 |  | 0.00 | -0.00 | 0.00 | 0.99 | .997 | |
|  | *F*(3, 306) = 4.91, *p* = .002, *R^2^* = .05 | | | | |  | *F*(3, 284) = 2.53, *p* = .058, *R^2^* = .03 | | | | |  | *F*(3, 1116) = 4.70, *p* = .002, *R^2^* = .01 | | | | | |
| *Note.* Including values that overlap multiple higher order domains (Humility and Face added to Conservation values, Hedonism added to Openness values). | | | | | | | | | | | | | | | | | | |

**Table S6**

*Uncentered Correlations for the Ten Basic Values (PVQ) with Relationship Quality* (Study 1, 3, 4)

|  |  | **Relationship quality** | | |
| --- | --- | --- | --- | --- |
|  |  | Study 1 *PVQ* | Study 3 *PVQ* | Study 4 *PVQ* |
| 1. | Universalism | .12^t^ | .24^***^ | .21^***^ |
| 2. | Benevolence | .22^***^ | .41^***^ | .27^***^ |
| 3. | Achievement | .07 | .10 | .01 |
| 4. | Power | -.05 | -.07 | -.08 |
| 5. | Hedonism | .18^*^ | .29^***^ | .16^*^ |
| 6. | Self-direction | .07 | .35^***^ | .13^*^ |
| 7. | Stimulation | .04 | .20^**^ | .11^*^ |
| 8. | Security | .18^*^ | .19^**^ | .09 |
| 9. | Tradition | .08 | .04 | .11^*^ |
| 10. | Conformity | .10 | .14^*^ | .12^*^ |

*Note.* ^t^*p* < .1, **p* < .05, ***p* < .01, ****p* < .001 (two-tailed).
